# Too afraid to go: fears of dignity violations as reasons for non-use of maternal health services in South Sudan

**DOI:** 10.1186/s12978-018-0487-6

**Published:** 2018-03-20

**Authors:** Sumit Kane, Matilda Rial, Maryse Kok, Anthony Matere, Marjolein Dieleman, Jacqueline E. W. Broerse

**Affiliations:** 1 KIT Royal Tropical Institute, Mauritskade 63, Amsterdam, 1092 AD The Netherlands; 2 Nossal Institute for Global Health, Melbourne School of Population and Global Health, University of Melbourne, Level 5, 333 Exhibition Street, Melbourne, VIC 3010 Australia; 3 Independent Consultant, Wau, Western Bahr el Ghazal South Sudan; 4 School of Public and Environmental Health, University of Bahr el Ghazal, Wau, Western Bahr el Ghazal South Sudan; 5 Athena Institute for Research on Innovation and Communication in Health and Life Sciences, Faculty of Earth and Life Sciences, VU University Amsterdam, Amsterdam, The Netherlands

**Keywords:** Maternal Health Services, South Sudan, Service Utilization, Dignity, Social Fears, Social Accessibility

## Abstract

**Background:**

South Sudan has one of the worst health and maternal health situations in the world. Across South Sudan, while maternal health services at the primary care level are not well developed, even where they exist, many women do not use them. Developing location specific understanding of what hinders women from using services is key to developing and implementing locally appropriate public health interventions.

**Methods:**

A qualitative study was conducted to gain insight into what hinders women from using maternal health services. Focus group discussions (5) and interviews (44) were conducted with purposefully selected community members and health personnel. A thematic analysis was done to identify key themes.

**Results:**

While accessibility, affordability, and perceptions (need and quality of care) related barriers to the use of maternal health services exist and are important, women’s decisions to use services are also shaped by a variety of social fears. Societal interactions entailed in the process of going to a health facility, interactions with other people, particularly other women on the facility premises, and the care encounters with health workers, are moments where women are afraid of experiencing dignity violations. Women’s decisions to step out of their homes to seek maternal health care are the results of a complex trade-off they make or are willing to make between potential threats to their dignity in the various social spaces they need to traverse in the process of seeking care, their views on ownership of and responsibility for the unborn, and the benefits they ascribe to the care available to them.

**Conclusions:**

Geographical accessibility, affordability, and perceptions related barriers to the use of maternal health services in South Sudan remain; they need to be addressed. Explicit attention also needs to be paid to address social accessibility related barriers; among others, to identify, address and allay the various social fears and fears of dignity violations that may hold women back from using services. Health services should work towards transforming health facilities into social spaces where all women’s and citizen’s dignity is protected and upheld.

## Plain language summary

Years of conflict have led to South Sudan having one of the worst health and maternal health situations in the world. While health services are being slowly rebuilt, many people, including pregnant women, do not use the available services. This study shows that women’s decisions to use available services were not merely about whether they were aware of risks involved in pregnancy and childbirth, or about whether the services were reachable, affordable or of good quality. We found that in South Sudan, the social norm is that a pregnant woman is expected to be well taken care of, and should be seen to be well taken care of, by her man and his family; the appearance of being well taken care of, socially dignifies the woman’s pregnancy. In view of this, a woman’s decision to seek care during pregnancy and childbirth also depended upon whether in the process of stepping out of her home to go and use services, her dignity as a pregnant woman could be maintained and protected - from the judging eyes of society, from other women in the health facility, and while interacting with health workers. Her decision thus also depended upon a complex trade-off she was willing to make between the benefits she thought the care would bring to her, and the potential risks to her social dignity. Explicit attention also needs to be paid to identify, address and allay the fears of dignity violations that may hold women back from using maternal health services in South Sudan.

## Background

South Sudan has one of the world’s worst population health indicators; for instance, the maternal mortality ratio stands at 789/100,000 live births [1], less than 30% of women are attended to by a skilled health worker; and the rate of institutional delivery assisted by a skilled birth attendant is less than 20% [2]. In post-conflict contexts, healthcare provision and improving population health outcomes is particularly difficult because of poor infrastructure, limited human resources, and weak stewardship [3–5]. In South Sudan too, the long drawn conflict has weakened the health system, and there are severe shortages of health workers and few well-functioning health facilities [2, 8]. De Francisco A et al. [6], drawing on the International Covenant on Economic, Social and Cultural Rights [7], argue that for public health programs to be useful to and to be used by the people they mean to serve, one “requires location-specific investigations” ([6], pg.19–20). In a recent review of maternal and child health policies in South Sudan, Mugo et al. [8] noted that “Informing policy with evidence requires acute sensitivity to local context”; in a subsequent paper, they further emphasize the need to “address the socio-economic factors that prevent women from using maternal health services” in South Sudan [9]. This paper presents the findings of such an investigation from South Sudan; it complements the recent work by Mugo et al. [10], Wilunda et al. [11], Lawry et al. [12] on barriers to maternal health in South Sudan.

This paper reports findings from a study done within the context of a project designed to support the Ministry of Health (MoH) of Western Bahr el Ghazal (WBeG) state of South Sudan to improve sexual and reproductive health (SRH) service delivery. There is robust evidence that “A health centre, intrapartum-care strategy can be justified as the best bet to bring down high rates of maternal mortality” [13]; the project, among other things, explicitly focused on improving access to and use of maternal health services. Within the operational research component of the project, a range of questions regarding SRH related behaviors and decision making were identified and studied; one of them being ‘why inspite of having maternal health services in the vicinity, many women still do not use the maternal health services?’

Findings from the broader study are reported in earlier papers which report how social norms and gender norms shape procreation decisions, birth spacing and family planning related decisions in South Sudan [14, 15]. Wilunda et al. [11] and Lawry et al. [12] in their recently published papers, elaborate upon geographical, financial, security and cultural barriers to the use of maternity services in South Sudan. These barriers also featured prominently in our study findings; however, we also identified other ‘social’ reasons why many women, do not use, or hesitate to use, the maternal health services currently on offer in Wau county of WBeG state of South Sudan. In this paper we focus on these ‘social accessibility’ [16] related barriers. We do so to highlight the importance of this important dimension of health services accessibility; in the process, we propose the notion of 'social accessibility' and contribute to extend the understanding of what all ‘health services accessibility’ should entail.

## Methods

A qualitative study was conducted; data was collected through focus group discussions (FGDs) and semi-structured interviews (SSIs) conducted with a variety of purposefully selected informants, as detailed in Table 1. Following sections further explain the sampling and recruitment principles and processes.

**Table 1:** Overview of study participants and data collection

Method	Profiles of study participants	Number of activities (number of participants)
FGD	Community members: Female 18–35 years (Not in union^a^)	1 (8)
	Community members: Female 18–35 years (In union)	1 (8)
	Community members: Male > 35 years	1 (8)
	Community members: Male 18–35 years	1 (8)
	Health workers	1 (6)
SSI with community members	Community member: Female 18–35 years (Not in union)	5
	Community member: Female 18–35 years (In union)	6
	Community member: Male 18–35 years	6
	Community member: Female > 35 years	6
	Community member: Male > 35 years	4
SSI with key informants	Traditional birth attendants	4
	Traditional leaders	3
	Health facility personnel	5
	State SRH managers	2
	NGO representatives	3

^a^Participants were either In Union or Not In Union at the time of the study. Relationship status is presented this way because in Wau people say they are married only if the relationship was formalised either in a traditional ceremony, or in the church – even if they cohabit. For convenience we use the terms married/unmarried in the paper

Topic guides for FGDs and SSIs were developed using de Francisco et al.’s (6) conceptual framework. According to the framework, individuals and social groups occupy positions of relative advantage or disadvantage with respect to their access to resources (social and material), within overlapping spheres of influence: the household, community, larger society, and the political environment. Individual’s and social groups’ position and relations in these overlapping spheres of influence shape their SRH related decisions and actions. Topic guides for community members included questions exploring people’s expectations from, and reasons for (non-)use of maternal health services. The topic guides for health and other workers included questions on the same lines, but with a view to explore their perspectives on the (non-)use of maternal health services. The FGD and SSI topic guides for community members were prepared in English and translated into the local language, Wau Arabic. The topic guides were defined further during the initial stakeholder workshops, pre-tested in the study site, and were adapted iteratively as the study progressed.

### Study sites

The study was conducted in Wau County of WBeG State of South Sudan. While South Sudan is home to more than 50 ethnic groups, in WBeG, the Fertit, an agriculturalist people, predominate. Two locations in Wau County were selected based on the homogeneity of the residents (all Fertit). Both locations were within walking distance of functioning maternal health services - this was important as health service coverage (geographical) is poor in many parts of WBeG. In both the locations, maternal health services were provided in a primary care facility staffed by one clinical officer, one nurse, 1–2 midwives and a pharmacist. In both facilities, the staff were a mix of locals, and returnees who originally hailed from WBeG. The two locations represented two different settings in Wau County – Wau town and the other a rural area. However, in both settings the socioeconomic situation was similar, with most people engaged in subsistence farming or informal manual labour. The assumption behind choosing these two locations was that perhaps within the same ethnic group, depending on the setting, the decisions and decision-making processes around whether or not to use maternal health services, might be moderated differently.

### Sampling, recruitment of study participants and data collection

Details of study participants are presented in Table 1. Community members were purposefully selected with the assistance of village elders, health workers from a local NGO and the county health department. The assistance was limited to guiding the researchers to the village and to making introductions; the actual selection was done by the researchers themselves. Amongst community members, only those of age 18 years and above were included in this study. We purposefully categorized participants into those between 18 and 35 years and those above 35 years with the assumption that the two age groups might have different health seeking behaviors.

Data collection began with FGDs amongst community members, followed by SSIs to obtain more in-depth understanding. FGD participants were homogenous in terms of ethnicity, age and marital status, yet diversity was sought in terms of social and economic status (criteria included ownership of assets like bicycles, and level of education). Health facility personnel responsible for maternal health in facilities close to the study sites were included as participants. Individuals with active maternal health related role within the county and state health system i.e. traditional leaders, traditional birth attendants, SRH service managers, and representatives of NGOs working on maternal health, were also included as key informants.

Data were collected from October 2014 to April 2015, over 3 visits to Wau. FGDs and interviews with community members, traditional leaders and traditional birth attendants were conducted by research team members who hailed from the study area, were fluent in Wau Arabic, and had experience with conducting qualitative research. Data were collected till analytical saturation was reached, and no new insight emerged; this was possible to assess, as at the end of each day of data collection, the research team debriefed and discussed the emerging findings. In total 5 FGDs (with 38 participants) and 44 SSIs were conducted.

### Data analysis

SSIs and FGDs were digitally recorded, translated from Wau Arabic into English (where applicable) and transcribed verbatim. An inductive thematic analysis of the transcripts was conducted [17]. Analysis began with an initial thorough reading of transcripts by three researchers (SK, MR, MK) to identify broad themes about the reasons for use or not of maternal health services. The guiding principle in this process was to identify the various reasons that were important to participants and to ascertain that the chosen themes captured the main aspects of participants’ reasons behind using or not using SRH services. The next step involved moving from these themes to an interpretation of the broader significance of and meanings attached to these themes, and the implications of these themes; in parallel, and iteratively through this process, the identified themes were reviewed, refined, and named. The NVivo 11 software was used to code all transcripts and to run queries on the dataset. Findings from the preliminary analysis were refined through follow up interviews with 2 participants in each study site (*n* = 4), one traditional leader, one local resource person, and through a workshop involving community health workers, health facility personnel and SRH services managers (*n* = 13).

### Ethical considerations

Informed consent was given by all study participants; for those who could not read, the consent form was read out to them and their consent was recorded. Confidentiality was maintained throughout, and steps were taken to anonymise the data and to minimise risk of accidental disclosure and access by unauthorized third parties. Since the study included questions about the local health services and the responsiveness of providers, special care was taken to ensure that identities of participants were not revealed to the local health workers. All participants were explicitly informed of their right to refuse to participate and to not answer questions they might find to be intrusive. Keeping in mind the possibility of some participants being reminded of traumatic experiences, medical referral services and counselling support were made available. No such situation requiring referral emerged during data collection or in the period after the study.

## Results

While the study did not dwell into the details of level and nature of knowledge about SRH matters, all community members – women and men across age groups, and all traditional leaders, recognized the benefits of modern maternity care, and were aware about the importance of antenatal care, institutional delivery, and to some extent, post-natal care. Reliable, state and county level data on availability, accessibility and utilization of maternal health services is not available in South Sudan. However, in the study area, services were available and accessible; and study participants indicated that they appreciated the presence of these services, and used these services. Issues related to geographical access, financial access, and perceived quality of care were reported as being important barriers to the use of services by our study participants. These barriers are important, however they are not the focus of this paper, and hence not presented and discussed here.

This section presents other social reasons why inspite of being knowledgeable about and having maternal health services in their vicinity, many women still did not use these services. Findings are presented as themes; three major themes emerged. The first theme presents how various social fears shape women’s care seeking. The second theme presents how women’s social expectations and social interactions around the act of visiting a health facility shape their care seeking behavior. In the third theme, women’s and society’s views about pregnancy are presented with a view to locate the findings of the first two themes in the local context, and to better explain them.

### Social fears

Women, both young and old, talked of fear and of ‘being afraid’ in some form or the other. They often did so without probing, indicating that the experience probably had wide relevance, and was an important feature of women’s interaction with the maternal health services, specifically of why women, used the maternal health services, or not. The importance of this cognitive process was acknowledged by professional informants too; although their observations were limited to and primarily referred to fears related to painful medical procedures and to the insecurity involved in the act of travelling to health facilities.

#### Fear of being embarrassed

Women were afraid of being embarrassed during the care encounter; in our study, this feeling had two broad facets. One related to not having enough money to cover the expenses incurred, and another related to not having one’s husband by one’s side.

Maternal health services in South Sudan are free in primary care facilities, although some user fees are levied in hospitals. However, the facilities in the study area often did not have enough supplies and drugs; patients were asked to buy these from private pharmacies. In Wau, people had to spend money to buy goods (soap, cloth, cotton, medicines etc) that are needed when delivering in a health facility, for transport, for stay if one were from another place (as is often the case around Wau), and also to pay for fees (including for informal payments to health workers). Not having enough money was clearly cited as a reason for not using services by both men and women. As the following quote illustrates, one of the underlying mechanisms through which not having money also shaped care seeking decisions, was that women were afraid that if they were to be asked to pay, and they did not have enough money on them, they would be shamed or even be belittled.

*“It might be money, some things go back to the economy, maybe there is no money and she is afraid that when she goes they will charge her a lot of money.” [Woman under 35, Not In Union].*

Another underlying mechanism through which not having money affected care seeking decisions, was that women were afraid that if they did not have enough money to pay for the expenses incurred at the hospital, they might not be allowed to return home. This led some to not only not use the hospital facilities, it also led them to turn to (and often to prefer) the services offered by traditional birth attendants (TBAs). Unlike hospitals, TBAs were flexible; they did not necessarily expect cash, and could be paid in instalments, over a longer period of time.

*“TBAs can wait even for a year for the women to pay them, but if you go to the hospital and you don’t pay they won’t let you go home, so women fear.” [FGD, Women above 35].*

Women who were widowed, or did not have a husband, or whose husband was away, or had been abandoned the husband, or had no family to support them, were afraid that health workers would ask them about their husbands, and would insist that their husbands be present. In South Sudan, a pregnancy is a matter of pride, and it is important that it is dignified by and seen to be valued by the man and his family. It is deeply embarrassing to women if they are seen to be on their own, and with no man to dignify their pregnancy. Women fear this embarrassment, and instead of going to health facilities, prefer to stay at home to avoid the embarrassment.

*“She is also afraid that they might tell her to bring her husband … and the man is not there. Because of fear they stay at home” [Woman under 35, Not in Union].*

For such women, as was often the case, not having enough money, further amplified the problem. They were particularly worried that if they did not have enough money on their person, the health workers might ask them to bring their husband, further exposing them to embarrassment.

#### Fear of being ill-treated

There is a large body of literature from low and middle-income countries which documents ill-treatment of patients by workers in health facilities. To some extent, and linked to the fear of being embarrassed, women in the study community were also afraid of the midwives being rude to them. In the following quote, a young woman points out how some women are so afraid, that they would rather deliver at home, inspite of knowing well that to do so, is dangerous.

*“The people who do not want to go to the hospital are people who are afraid. They fear delivery, and fear that the midwives will be rude to them. So that is why they don’t go to the hospital but still deliver at home .. (even when they know that it).. is dangerous.” [Woman under 35, Not in Union].*

Senior health workers and SRH service managers, recognised this situation. They were well aware of, and felt ashamed about the poor attitudes of some of their staff. Privately, some expressed frustration at the situation – pointing out that the shortage of health workers in the area meant that hey had very little room to reprimand and discipline errant health workers.

*“Yes I do agree, this situation is very embarrassing … some midwives are verbally abusive and have bad attitudes. Some women will prefer not to come back to the hospital because of the maltreatment.” [Health Facility Personnel - Manager].*

#### Fear of being denied services

Many steps are being taken to improve maternal health services in South Sudan; for instance, to improve the continuity of care, a paper card is issued to every pregnant woman. In this card, health workers record the progress of the pregnancy and the pregnant woman’s medical situation. We found that health workers diligently use these cards, and impress upon women the importance of carrying these cards when they visit health facilities; most women also understood the importance of these cards. However, it was these very cards that paradoxically appeared to hinder the use of maternal health services. Some women could not afford these cards (approximate price = 0.25 EUR), and therefore hesitated to visit the health centres. We found that many women lost their cards, had their cards torn, or soiled; as the following quotes show, women in such situations were afraid of being reprimanded by the health workers, and denied services.

*“This will affect you, if you have a child (are in the process of delivery) and you do not have a follow up card no one will accept you even the trained midwives they will not assist you. Even the hospital will not accept you.”[Woman under 35, Not in Union].*

*"If the midwife finds that you do not have a hospital card, she will tell you that she cannot go to you because you did not go for checkups. If a crime comes to me, what will I say? I will not go to you. We have this kind of situations here." [FGD, Women over 35].*

These quotes also illustrate how the way these check-ups and cards related processes were implemented in practice; perhaps unwittingly, these service delivery improvement processes, paradoxically gave some women the impression that not carrying these cards, or not attending earlier antenatal check-ups, was akin to committing a crime. An impression that seemed to be enough to make some women afraid, and to not use maternal health services.

#### Insecurity related fear

Poor rule of law is a problem in much of South Sudan. The state apparatus is unable to protect people from antisocial elements, including but not limited to ethnic militias. The prevailing insecurity featured prominently in both men and women’s explanations for not using health facilities. People were afraid of being accosted on the way to the health facilities at night, but also during the day.

*“If labour pains start at around 2 am, and there is no way to go to the hospital, and there is no transport, and you fear criminals on the way.” [Woman under 35, Not in Union].*

*“There is no transport, so people fear to move at night to go to the hospital and people can attack you on the way” [FGD, Men under 35].*

The health workers, the healthcare managers all admitted that this was a major problem. They acknowledged the circumstances and they recognized people’s fears as understandable, pointing out that this was the price society paid on a daily basis for the chronic insecurity and unrest.

*“And for people to access services there must be security, people should have peace of mind that if I walk five kilometers, I will go and come back without any problem. So one of the factors is .. if I go there and I feel threatened (on the way to getting to the facility), it will affect the utilization.” [NGO Representative].*

The findings above reveal that a variety of social fears also shape decisions around seeking maternal health care. In the discussion section, these fears, and the social processes driving them, are discussed in view of the theoretical insights on ‘social fears’.

### Dignity expectations not being fulfilled

In the study community, as in all communities in South Sudan, pregnancy is a matter of personal pride for women. It is something to be celebrated and dignified by the man’s family. As the following quotes from an FGD among men illustrate, it is expected that a pregnant woman is treated nicely and is seen to be so too in society, particularly when she ventures out of the house and into public spaces.

*“When you (a pregnant woman) get up to go to the health center, the culture and traditions are like … the shoes on your feet and the clothes … when you want to leave your house, you need to take a shirt and wear (good clothes).” [FGD, Men over 35].*

Being able to dress nicely, and to be presentable in public spaces like the clinic, was very important to women. It was important to the extent that if they did not have soap to bathe and did not have a clean dress to wear, they would rather not go to the clinic – inspite of knowing well the importance of the antenatal, natal or postnatal visits.

*“When they get pregnant, they want their husbands to buy them new dresses, new shoes, to braid their hair … and to give her money … then after … that is when you leave home and go (out into public places, like the health centre).” [Woman under 35, Not in Union].*

Women whose husbands were either away, or who had been abandoned by their husband, or had nobody to provide for them, would rather not be seen in public in an unpresentable state. Appearing disheveled and uncared for would give people an impression that this was someone whose pregnancy was not being celebrated and dignified by the family. Women in such circumstances would rather forgo care, than open themselves to dignity violations. While reliable data are not available, many women in the study community, and in South Sudan at large are in such a situation.

### The ‘pregnancy’ - for the man’s family, and also the man’s responsibility

In some ways linked to all of the above themes, and in many ways shaping women’s care seeking decisions and actions, albeit at a cognitively different level, is the status and role of women in the local society, and how women see themselves within and interact with these social arrangements. We found that women’s role in society is seen to primarily be about bearing children for the man’s family. The entrenched social norm is that women must bear as many children as the man and his family members wish; this norm relates to the idea that children replace the dead, and they allow inheritance and the continuation of the man’s family name. The following two FGD interactions illustrate the local social reality and how men and women relate to it. The first interaction below, in an FGD among young women, illustrates how women see and experience their situation and role in the man’s family; it also highlights how not bearing children as demanded by the man and his family, incurs the risk of being abandoned by the man.

*Participant 1: “If you are married and already living with your husband and do not have a child, the husband can leave you and tell you to go back to your family.”*

*Participant 2: “His relatives will come and argue that why you are not getting pregnant …the man’s relatives will complain why is this woman brought and eating our food for free if she is not going to deliver children.”*

*Participant 1: “The relatives will tell the husband to leave you and go and get another woman who can have children.”*

*Participant 3: “Or the (man’s) relatives themselves will go and get a wife for their son.” [FGD, Women under 35].*

The second interaction below, in an FGD among men, men nonchalantly discuss their inalienable claim on the woman’s womb and her fertility potential. They refer to the woman as ‘our’ wife – it signifies not just the man’s claim, but rather the family’s, for they have bought her, and brought her into the family, with the purpose of bearing children for the family. The discussion shows how the man, and the man’s family not just expect the woman to give them children, her not doing so, is considered sufficient grounds to abandon her and replace her.

*Participant 1: “Because this is* *our* *wife,* *we* *married her with money. Of course, marrying a woman is like business … is like business. Meaning that if you start a business you must profit from it.”*

*Participants 2,3,4 (In chorus): “Yes, Yes.”*

*Participant 1: “And if you take a woman with money and she does not give you children, that is not good.”*

*Participant 2: “Yes, the family … if a woman is pregnant your family is happy.”*

*Participant 3: “They will say that this woman is now giving birth replacing the person who had died … the one inside now is in place of the person who had died. The family will be happy.”*

*Participant 4: “Like … this is my son here. He married a woman. His wife is bearing children. I will be happy. Some people meet me and say … Oh Peter! Your son’s wife delivered. I’ll be happy... But if my son married a woman and she does not bear a child, eating the ‘Asida’ (food) for free, I will not be happy.” [FGD, Men under 35].*

Women’s awareness of their status in the man’s household, and their cognizance of the social reality that they had been brought (even, bought) into the man’s household to bear children, appeared to result in an ambivalent attitude towards pregnancies generally, including towards their own pregnancy. This layered sociality also shaped women’s approach towards using maternal health services. Women seemed to view the (unborn) child as the man’s family’s, and also seemed to view the process of using maternal health services as not being about their own health, but rather being about the health of the (unborn) child, and thus the responsibility of the man and his family, and not their own.

*Facilitator: “Some women do not go to the hospital what makes them not to go?”*

*Participant: “Sometimes the man says he does not have money that was why she could not go for check-up. So, she decides not to go and if the baby dies it is a loss for her husband’s family and not her family.” [FGD, Women under 35].*

That having been said, this approach to pregnancy and maternity care was not universal. Many women, even when their husband did not provide them with the money, still used antenatal and delivery services; they did so through raising money from other sources. In such situations some women also resorted to using the services offered by traditional birth attendants who charged less, were open to being paid in kind, and to being paid in instalments.

## Discussion

Consistent with Wilunda et al. [11] and Lawry et al’s [12] studies from South Sudan [11, 12], and the global health services literature [18, 19], our study also found that women do not use the services, if they don’t feel the need, if services are inaccessible (geographically and financially), if they perceive services to be of poor quality, and if they do not have confidence in the competence of providers. Instead of repeating what Wilunda et al. [11] and Lawry et al. [12] have reported in detail, in this paper we choose to focus upon an important, and often insufficiently reported dimension of access – ‘social accessibility’ [16]. In the following discussion section, we draw upon our insights about the context of South Sudan, theoretical insights, and empirical findings from literature, to discuss our findings about women’s views on pregnancy, women’s dignity expectations, and social fears around the act of seeking care. In doing so, we also extend the conceptual understanding of what constitutes accessibility of health services, to include the notion of ‘social accessibility’; we make a case for inclusion of this understanding when studying access to health services and when intervening to improve access to services.

We begin with a discussion on what we consider a meta construct shaping women’s thinking about pregnancy, and their approach to dealing with it. This background sets the stage to further discuss our findings within two broad and linked theoretical frames: social fears, and social dignity and its violations. Throughout the process, implications are drawn for public health policy and practice in South Sudan, and where appropriate, beyond; in the process, we add to and nuance this body of knowledge on multiple fronts. In doing so, the importance of ‘location specific investigations’ recommended by De Francisco et al. [6] and others [8], is reiterated.

### Carrying a child for someone else

Women’s ambivalent, sometimes even uncaring attitudes towards pregnancies, including their own pregnancy, need to be understood better. Oyewumi’s work [20] on family structure and social relations in many African societies, provides a useful frame to understand this ambivalence; according to Oyewumi, in many African societies, the family unit is a “consanguinally-based family system built around a core of brothers and sisters-blood relations, wherein the spouses are considered outsiders and therefore not part of the family” [21]. This is unlike the Western family structure of a conjugally-based family built around a couple. In many communities of South Sudan, including in WBeG, the family unit is a consanguinally-based unit, and the woman remains an outsider whose primary role is to bear children for the man’s family. This family structure and the social norms that accompany it, in some ways explains why many women viewed the child they were carrying as someone else’s and for someone else, and its care was thus also seen by them as being someone else’s responsibility – the someone else being the husband and his family. We argue that these social relational arrangements shape how women view and relate to their own pregnancy, and also what they are willing to do or not do, about it. These social-relational arrangements thus constrained women’s use of maternal health and SRH services. Pregnant women seemed to also somehow use these social arrangements as a rationale to justify their actions or inactions, which they well knew as being not good for the health of the unborn.

#### Social fears

‘Fears’ of different kinds emerged as a key concern shaping the non-use of maternal health services. Findings show that the broader theme of ‘social fears’, is constituted by and subsumes sub-themes which reflect a wide range of social processes: unequal power relations between providers and patients, professional control over the patient-provider interaction and the linked sense of undermined agency, and the broader insecurity in region and country. The latter being a fear, but also perhaps an enabler of other fears; insecurity tends to undermine use of services through, among others, pushing up opportunity costs of accessing services. These sub-themes not just represent different facets of social fear experienced by women when interacting with or contemplating the use of maternal health services, they also help explain how the fear experience is mediated in a number of specific social contexts, and shapes women’s decisions to use maternal health services.

Drawing on Tudor [22], we argue that the many fears articulated by women are perhaps best understood as ‘social fears’, where they relate to and are shaped by the attributes of the social worlds individuals inhabit. Social fears may be explicit or tacit, big or small, but they pervade all aspects of our lives and are a key feature of many social situations and interactions; Tudor argues that these fears featuring within social situations “have complex ramifications for the ways in which we live our lives” [22]. If one’s environment, and social interactions repeatedly signal that a certain kind of activity or interaction is unpleasant, painful or dangerous, and could lead to trouble, and if these signals are experienced by others in one’s environment, then this provides the conditions for fear to emerge for that particular activity or social interaction. Such fears, once established, have a powerful effect on the actions of individuals. Women who harbor fears, however big or small these fears might be, are unlikely to easily use the maternal health services on offer; furthermore, if sufficient number of women harbor such fears, it influences the thinking of others around them. Evidence shows that experts’ and professionals’ control over many areas of human activity, like healthcare, can promote fear also beyond the worries related to the direct consequences of specific actions and interventions – including, as in this case, rude/abusive behaviour [23, 24]. It can partly also explain why many women turn to and prefer traditional birth attendants.

Discussing the care experience of study participants within a relational context can help better understand the point regarding care providers. In South Sudan, including in Wau, the relations between the locals who stayed behind during the war, and the returnees who had fled to the erstwhile Sudan during the war, are tense. The returnees have brought valuable knowledge and skills back with them - a large proportion of health care providers are returnees. However, many returnees, because they have been away for decades, do not identify sufficiently with the locals, and tend to be judgmental of the locals. While on one hand the locals welcome and appreciate the returnees, they are keenly aware of the subliminal othering effected by the returnees. This, together with the underlying feelings of resentment amongst those who stayed, towards those who went away, makes the interaction between the locals and the returnee providers, complex, and presents a fertile ground for manifestation of fears. That said, the social dynamics between the returnees and locals was not the focus of our study; further investigation is needed to understand it and its possible public health implications.

#### Social dignity and dignity violations

Women’s decisions to step out to seek care are the results of a complex trade-off they make or are willing to make between potential threats to their dignity in social spaces, their views on ownership and responsibility for the unborn, and their fears regarding the care encounter. Many of the fears expressed by women are perhaps better understood in terms of them being afraid of their dignity being violated in the various interactions entailed, and the spaces traversed, in the act of seeking care. We draw upon the notion of social dignity [25, 26], the social and cognitive processes around its maintenance and its violations, to better understand our major findings. Social dignity as a concept refers to the idea that in every social interaction, the dignity of one or more participant or bystanders, can potentially be upheld, promoted, threatened or violated. What entails social dignity, and perceptions of when it is upheld, threatened or violated, is socially constructed – it depends on the norms and traditions of a particular community or society. The socially constructed nature of social dignity means that people in every society have tacit knowledge of how to assess it, when to expect its violation, and when to expect its upholdment. For a social interaction to become a dignity violation does not necessarily require explicit action or words being said, it can simply be an act of interpretation by the one whose dignity is at risk, or by others involved in the interaction, including the bystanders.

Consistent with the global literature on dignity and healthcare services [23, 25, 27, 28], women in our study reported being afraid of how they might be treated by healthcare workers in their care encounters. The mechanism underlying this fear of being abused, being reprimanded, being shamed, or being belittled during the care encounter, was fear of their social dignity being violated. This concern with social dignity, and its risk of being violated was however not restricted to the care encounter alone; women accorded great importance to, and were concerned about how they were seen in public spaces, be it on the way to the health facility, or in the health facility premises. These spaces were arenas for social interactions where community members, primarily women, asserted their social standing in relation to others. Those pregnant women who could not dress up, and look nice and be seen as being well taken care of, often preferred to stay at home and not seek care, to protect themselves from the judging eyes of other women and society at large, thereby guarding their social dignity. Not having the means to meet the social expectations, socially enforced or imagined, was a reality for most women in the study community. If a woman is in a position of vulnerability, she is more likely to interpret even a relatively minor social slight as violation of her social dignity [25, 26], and as discussed earlier, if the woman happens to see the process of making herself vulnerable to such a violation as not being in her benefit or her responsibility, she will avoid even initiating the process of seeking care. Jacobson [26] argues that in difficult circumstances, some, particularly those who are most disadvantaged and vulnerable, may be so worn down by the constant micro insults and violations of their social dignity, that they may isolate themselves and avoid social interactions as much as possible – this manifests as “a reluctance to seek help or access resources, passivity or ‘learned helplessness’”. Many women in our study community, and in many parts of South Sudan, are in such a position.

### Limitations

The study has some limitations. There was a possibility that people would only give socially desirable answers with the view to not antagonize health workers. These constraints were anticipated, and steps were taken to loosen these constraints. Data collection with community members was done by researchers who hailed from the local community, were independent, and were in no way related to the NGOs delivering services. They also knew the local culture well, and took due care to ensure frank and open interactions. Visits were made to the study sites before the actual data collection to meet the villagers and the elders – to explain the nature of the study, to seek the village’s agreement for our presence, and to reassure them that confidentiality will be maintained. We observed that participants were eager to participate and happy that they were being heard. Throughout data collection, the interactions with participants were frank and candid; it makes us confident about our study findings.

## Conclusions

Barriers related to geographical accessibility, affordability, and perceptions (need and quality of care) hinder women from using maternal health services, and these barriers need to be addressed. Through this paper we show that it is equally important to also explicitly consider, and address barriers related to social accessibility – our findings highlight how social fears and fears of dignity violations may hold women back from using maternal health services. We argue and conclude that interventions to improve accessibility of maternal health services should have features that protect women from dignity violations in the care encounters, and should make and shape health facilities into spaces for dignity promotion, for one and all.

Making health facilities into spaces for dignity promotion requires the explicit embedment of dignity considerations in all aspects of service organisation, across provider-patient encounter settings, and across the health facility as a social space. Specifically, and drawing on Jacobson [29], among other things, this could entail the development of a local diagnostic tool which allows service users and practitioners to jointly reflect on “their own positions of vulnerability and antipathy, and on the nature of the gestures, interpretations, and responses that constitute dignity violation in their own settings, using this exercise to change the dignity dimensions of their interactions”, and of the spaces in which these encounters occur. Doing so will not only draw women to health facilities, it can also contribute to broader social cohesion and social development by signalling social equality regardless of ethnicity, social and economic status.

Dignity violations in the healthcare encounter and in the health facility space are but reproductions and manifestations of the structural inequalities prevalent in a society. These structural problems require structural solutions at the societal level. While these solutions are beyond the purview of health workers, public health programs and health workers can contribute to triggering social change by both, changing things in the health facility and creating exemplar islands of possibility, and also by actively making common cause with those who are working on promoting social justice, rule of law, and tackling gender and social inequalities in the society.

### Summary

Women will use available maternal health services, if they feel the need for the services, if the services are geographically and financially accessible, and if they perceive the services to be of good quality. This study, adds that while these conditions are necessary, they may not be sufficient for women to step out of their homes to use services.

This study argues that the act of seeking care is a social act which entails many social interactions, in a variety of social spaces eg. the neighborhood to be traversed, the waiting area of the health facility, and the care encounter setting. And that, in the many potential social interactions that occur while traversing these spaces, depending on the local social norms, the woman’s dignity may potentially be upheld, promoted, threatened or violated.

This study contends that women’s decisions to use available services are shaped by a complex trade-off making process, between, on one hand, their beliefs about the importance of using services, and on the other, their fears about their dignity being violated during the interactions entailed in the act of seeking care.

This study concludes that policy makers should pay explicit attention to the embedment of social accessibility related considerations in all aspects of health services organisation, across provider-patient encounter settings, and across the health facility as a social space.

## Abbreviations

FGD: Focus group discussion
MoH: Ministry of Health
SHARP: South Sudan health action and research project
SRH: Sexual and reproductive health
SSI: Semi structured interview
WBeG: Western Bahr el Ghazal State

## References

[1] WHO (2015). Trends in maternal mortality: 1990 To 2015: estimates by WHO, UNICEF, UNFPA, World Bank Group and the United Nations Population Division.

[2] Ministry of Health (2012). Republic of South Sudan. Health Sector Development Plan 2012–2016.

[3] Roberts B, Guy S, Sondorp E, Lee-Jones L (2008). A basic package of health services for post-conflict countries: Implications for sexual and reproductive health services. Reprod Health Matters.

[4] World Bank (2011). World development report 2011. Overview: Conflict, security, and development. The World Bank.

[5] Haar RJ, Rubenstein LS (2012). Health in post conflict and fragile states.

[6] de Francisco A, Dixon-Mueller R, d’Arcangues C (2007). Research issues in sexual and reproductive health for low- and middle-income countries. Global forum for Health Research and world health Organization.

[7] United Nations General Assembly. Right To Health: International covenant on Economic, Social and Cultural Rights. January. 1976.

[8] Mugo N, Zwi A, Botfield JR, Steiner C. Maternal and child health in South Sudan: priorities for the Post-2015 agenda. Sage Open 2015; 10.1177/2158244015581190.

[9] Mugo N, Agho KE, Zwi AB, Dibley MJ. Factors associated with different types of birth attendants for home deliveries: an analysis of the cross-sectional 2010 South Sudan household survey. Glob Health Action. 2016; 10.3402/gha.v9.29693. 10.3402/gha.v9.29693 PMC5055614 27473675

[10] Mugo N, Dibley MJ, Agho KE (2015). Prevalence and risk factors for non-use of antenatal care visits: analysis of the 2010 South Sudan household survey. BMC Pregnancy Childbirth.

[11] Wilunda C, Scanagatta C, Putoto G, Takahashi R, Montalbetti F, Segafredo G, APl B. Barriers to institutional childbirth in Rumbek North County, South Sudan: a qualitative study. PLoS One. 2016; 10.1371/journal.pone.0168083. 10.1371/journal.pone.0168083 PMC5158020 27977745

[12] Lawry L, Canteli C, Rabenzanahary T, Pramana W (2017). A mixed methods assessment of barriers to maternal, newborn and child health in Gogrial west, South Sudan. Reprod Health.

[13] Campbell OMR, Graham WJ (2006). Strategies for reducing maternal mortality: getting on with what works. Lancet.

[14] Kane S, Kok M, Rial M, Matere A, Dieleman M, Broerse JEW. Social norms and family planning decisions in South Sudan. BMC Public Health. 2016; 10.1186/s12889-016-3839-6. 10.1186/s12889-016-3839-6 PMC5120474 27876018

[15] Kane S, Rial M, Matere A, Dieleman M, Broerse JEW, Kok M. Gender relations and women’s reproductive health in South Sudan. Glob Health Action. 2016; 10.3402/gha.v9.33047@zgha20.2016.9.issue-s4. 10.3402/gha.v9.33047 PMC5129092 27900934

[16] Powell M (1995). On the outside looking in: medical geography, medical geographers and access to health care. Health and Place.

[17] Braun V, Clarke V (2006). Using thematic analysis in psychology. Qual Res Psychol.

[18] Say L, Raine R (2007). A systematic review of inequalities in the use of maternal health care in developing countries: examining the scale of the problem and the importance of context. Bull World Health Organ.

[19] Gabrysch S, Campbell OM (2009). Still too far to walk: literature review of the determinants of delivery service use. Pregnancy Child Birth.

[20] Oyewumi O. Gender Epistemologies in Africa: the gendering of African traditions, spaces, social identities and institutions. Basingstoke: Palgrave Macmillan; 2011. ISBN: 978–0–230–62345–3.

[21] Oyewumi O. Conceptualizing gender: the Eurocentric foundations of feminist concepts and the challenge of African epistemologies. In: Anfred S, Bakare-Yusuf B, Kisiangani EW, Lewis D, Oyewumi O, Steady FC (Eds) 'African Gender Scholarship: Concepts, Methods and Paradigms'. Council for the Development of Social Science Research in Africa, 2004. Dakar, Senegal. ISBN: 2-86978-138-5.

[22] Tudor A (2003). A (macro) sociology of rear?. Sociol Rev.

[23] Bohren MA, Vogel JP, Hunter EC, Lutsiv O, Makh SK, Souza JP, et al. The Mistreatment of Women during Childbirth in Health Facilities Globally: A Mixed-Methods Systematic Review. PLoS. Med. 2015; 10.1371/journal.pmed.1001847. 10.1371/journal.pmed.1001847 PMC4488322 26126110

[24] Chi PC, Bulage P, Urdal H, Sundby J (2015). A qualitative study exploring the determinants of maternal health service uptake in post-conflict Burundi and Northern Uganda. BMC Pregnancy Childbirth.

[25] Jacobson N (2007). Dignity and health: a review. Soc Sci Med.

[26] Jacobson N (2009). A taxonomy of dignity: a grounded theory study. BMC Int Health Human Rights.

[27] Freedman LP, Kruk ME (2015). Disrespect and Abuse of women in childbirth: challenging the global quality and accountability agendas. Lancet.

[28] Miller S, Abalos E, Chamillard M, et al. Beyond too little, too late and too much, too soon: a pathway towards evidence-based, respectful maternity care worldwide. Lancet. 2016; 10.1016/S0140-6736(16)31472-6. 10.1016/S0140-6736(16)31472-6 27642019

[29] Jacobson N (2009). Dignity violations in health care. Qual Health Res.

